# Therapeutic efficacy test in malaria *falciparum *in Antioquia, Colombia

**DOI:** 10.1186/1475-2875-5-14

**Published:** 2006-02-20

**Authors:** Silvia Blair, Jaime Carmona-Fonseca, Juan G Piñeros, Alexandra Ríos, Tania Álvarez, Gonzalo Álvarez, Alberto Tobón

**Affiliations:** 1 Grupo Malaria, Universidad de Antioquia. Calle 62 52-59, Laboratorio 610. Medellín, Colombia

## Abstract

**Objective:**

Evaluate the frequency of failure of eight treatments for non-complicated malaria caused by *Plasmodium falciparum *in patients from Turbo (Urabá region), El Bagre and Zaragoza (Bajo Cauca region), applying the 1998 protocol of the World Health Organization (WHO). Monotherapies using chloroquine (CQ), amodiaquine (AQ), mefloquine (MQ) and sulphadoxine-pyrimethamine (SP), and combinations using chloroquine-sulphadoxine-pyrimethamine (CQ-SP), amodiaquine-sulphadoxine-pyrimethamine (AQ-SP), mefloquine-sulphadoxine-pyrimethamine (MQ-SP) and artesunate-sulphadoxine-pyrimethamine (AS-SP), were examined.

**Methodology:**

A balanced experimental design with eight groups. Samples were selected based on statistical and epidemiological criteria. Patients were followed for 21 to 28 days, including seven or eight parasitological and clinical evaluations, with an active search for defaulting patients. A non-blinded evaluation of the antimalarial treatment response (early failure, late failure, adequate response) was performed.

**Results:**

Initially, the loss of patients to follow-up was higher than 40%, but the immediate active search for the cases and the monetary help for transportation expenses of patients, reduced the loss to 6%. The treatment failure was: CQ 82%, AQ 30%, MQ 4%, SP 24%, CQ-SP 17%, AQ-SP 2%, MQ-S-P 0%, AS-SP 3%.

**Conclusion:**

The characteristics of an optimal epidemiological monitoring system of antimalarial treatment response in Colombia are discussed. It is proposed to focus this on early failure detection, by applying a screening test every two to three years, based on a seven to 14-day follow-up. Clinical and parasitological assessment would be carried out by a general physician and a field microscopist from the local hospital, with active measures to search for defaulter patients at follow-up.

## Background

After the use of any antimicrobial, treatment failure or resistance occurs in a percentage of cases, due to the variants or genetic mutants. In *Plasmodium*, mutations appear with a frequency of 9.5-71.2 × 10^-10 ^mutations/site/year in coding regions [1] and with a frequency of 1.5910 × 10^-4 ^mutations/site/year in microsatellites [2].

The *in vitro *evaluation of resistance of *Plasmodium *to an antimalarial drug consists of assessing growth inhibition when exposed to different concentrations of the drug, and several methods have been described [3-6]. The *in vivo *evaluation, or therapeutic efficacy test (TET), measures the clinical and parasitological response of malaria patients to a specific antimalarial treatment. The clinical response is indicated by the clearance of symptoms and signs, and the parasitological response by the clearance of blood parasites, after oral administration of recommended doses of one or several drugs, under supervision of the sanitary staff, and with a follow-up during a determined period (usually, 7 to 28 days). The TET evaluation protocol, approved by the World Heath Organization (WHO) in 1998 [7], has been used in many South-American studies. In this protocol, TET has been classified into three categories: early treatment failure, late treatment failure and adequate response. This treatment failure refers exclusively to inability to be cured of malaria, not to the inability to prevent the disease when the drug has been used in a prophylactic fashion. Treatment failure has been described in two out of the four species of *Plasmodium*, which affect humans: *Plasmodium vivax *and, especially, *Plasmodium falciparum*; the latter has shown resistance to many antimalarial drugs [8].

In Colombia, between 1961 and 2003 (Table 1), the non-weighed mean of treatment failure in falciparum malaria was 66% for CQ (13 reports), 22% for AQ (9 reports) and 15% for SP (14 reports). In recent publications, the failure of the malaria control programme in Colombia has been described and the causes analysed [9-11]. The geographic, environmental, socio-demographic, economic characteristics and general malaria morbi-mortality, in Antioquia, Urabá and Bajo Cauca regions as well as in El Bagre and Turbo municipalities have been detailed by others articles [10,12-15]. Demographic and epidemiological changes ocuring in Colombia during the XXth century have been reviewed and detailed elsewhere [16]. These reports constitute an integral reference framework to interpret the antimalarial treatment failure and resistance and to outline policies and programmemes to reduce their effects.

**Table 1:** Frequency (%) of failure *in vivo *in antimalarial treatment; Colombia, 1961–2003 (1)

**Place, Drug **(2)	**CQ**	**AQ**	**SP**	**Reference**
Colombia	82,0			Walker 1968 [38]
Colombia (4)	16,4	28,3	34,7	Blair 1986 [39]
Colombia (5)			24,0	(quoted in Ravreda 2003) [40]
Colombia (5)			25,0	(quoted in Ravreda 2003) [40]
Colombia *in vitro*	96,0	3,3		Espinal 1985 [41]
Antioquia (state)			34,0	(Restrepo quoted in Ravreda 2003) [40]
El Bagre (Ant ioquia)	82,0	38,0	10,0	Flórez 1988 [42]
El Bagre (Ant ioquia)	71,0	12,0	15,0	López 1999 [27]
Turbo (Ant ioquia)	97,0	7,0	13,0	Blair 1999, 2001 [28, 29]
Zaragoza (Ant ioquia)	67,0	3,0	9,0	Blair 1999, 2002 [28, 30]
Chocó (state)	52,0			Comer 1968 [43]
Quibdó, (Chocó)	44,0			Osorio 1999 [44]
Quibdó, (Chocó)			6,0	Osorio 1999 [44]
Tadó, (Chocó)		27,0	16,0	(González 2002 quoted in Ravreda 2003) [40]
Cali (Valle)	78,0			Castillo 2002 [45]
Buenaventura (Valle)			3,0	Méndez 2002 [46]
Tumaco (Nariño)	70,0	54,0		Flórez 1988 [42]
Tumaco (Nariño)	40,0	18,0	0,0	Flórez 1988 [42]
Tumaco (Nariño)		50,0	15,3	González 2003 [31]
El Charco (Nariño)		0,0	0,0	González 2003 [31]
Orinoquía y Amazonía			(3)	Espinal 1985 [41]
*Average*	66,3	21,9	14,6	
*Mean*	70,0	19,9	14,6	

(1) Response evaluated in patients (*in vivo*), except when the contrary has been indicated (*in vitro*). Duration: approximately from 1961–2003. Blank cells: It was not possible to find information.

(2) AQ amodiaquine, CQ chloroquine, SP sulphadoxine-pyrimethamine, CL clindamicina, MQ mefloquine.

(3) Espinal's report 1985 [41]: it does not mention the amount of failure to SP; it only refers to 3 cases in Orinoquía and 9 in Amazonía.

(4) AQ-CL: Blair 1986 [39] reports one study with 3,8% of failure.

(5) MQ-SP: failure of 0% in 1982 and in 1985; also failure of 0% in Botero *et al*. 1985 [47]. For MQ: 8.3% of failure in Amazonía.

(6) AQ-SP: González report [31], 11.0% of failure in this place.

On this background, this report: a) collects and summarizes the available information about treatment failure in Colombia; b) presents the results of the evaluation of TET for various treatment schedules, between 2000 and 2004, in the municipalities of Turbo (Urabá region), El Bagre and Zaragoza (Bajo Cauca region), in the north-west and north-east of the state of Antioquia, Colombia; c) collects and summarizes information about use of the combination AQ-SP for the treatment of non-complicated falciparum malaria; and d) discusses the creation of an epidemiological surveillance system of antimalarial treatment failure as part of the malaria programmeme operating in the country.

## Materials and methods

### Design of studies

The TET with eight schemes was assessed in a sequential way, between 2000 and 2004. All throughout the study period at least two simultaneous groups were being studied. Patients were randomly assigned a specific treatment as they arrived; this was guaranteed by a blind lottery (tombola). The group of studies constitute an experimental design (randomized clinical trial or clinical controlled study) [17] with eight equal groups, where the "exposition factor" (explicative variable) has been constituted by each treatment, and the "effect" corresponds to the therapeutic response.

### Site of study

Urabá and Bajo Cauca, two regions of the Antioquia state, generate 90% of malaria cases of this state. Urabá is located on Urabá Gulf in the Caribbean sea, near the borders with Panama; the territory has mostly a warm climate. Antioquian Bajo Cauca is located north of the state and has mostly a humid-very humid warm climate [18-20].

The Urabá region extends over 11,671 km^2^, while the Bajo Cauca region extends over 8,498 km^2^. In June 2002, Urabá had 463,496 inhabitants and Bajo Cauca 219,951. The Urabá population density was 36,4 inhabitants/km^2 ^in 1998 and 39,7 in 2002; in Bajo Cauca it was 21.5 in 1993 and 25.8 in 2002 [18-20]. In these two regions, the whole population is exposed to risk of malaria. Most of the inhabitants of these two regions are "mestizos" (creoles). In Urabá, there are few indian comunities: Kunas (Tule), Emberas (Katíos) and Zenúes [19]. From 1995 to 2000, the displaced population was 25,000 in Urabá and 5,000 in Bajo Cauca [18] but this was notorious between 2001 and 2005 [21].

In Turbo, 60% of the population lives in rural areas (villages or small towns), while in El Bagre only 38% lives in the rural area. The age structure of the two populations is similar [22]. In 2002, 8.38% of the Antioquia population lived in Urabá and 3.98% in Bajo Cauca. On the other hand, Turbo had 25.62% of Urabá population while El Bagre had 27.20% of Bajo Cauca population.

Outwash mining is the principal economic activity of Bajo Cauca, followed by cattle farming and, to a lesser degree, rice and sorghum cultivation [20]. In Urabá, the banana growing industry is the main activity, mostly concentrated in the central zone, followed by cattle farming in the northern part and timber exploitation from natural forests in the southern zone. There has been substantial migration of populations over the past 20 years, for economic and socio-political reasons. These migrations have contributed to the malaria problem, spreading *P. falciparum *resistant to chloroquine and other drugs, as was the case on the borders of Thailand with Cambodia and Myanmar [8].

The mean Annual Parasite Index (API) was 39 cases per 1,000 inhabitants in Turbo (1995–2000), 134 in El Bagre (1995–2000) and 33.5 in Zaragoza (1998–2000). For the period 2001–2003, the API was 19.5 in Turbo, 37.5 in El Bagre and 34.5 in Zaragoza (DSSA data). These data, with an API above 10/1,000, confirm the high-risk for malaria transmission in these regions.

### Sample design

The calculated size sample was 42 for each of the eight treatment schedules, and for each region selected (Turbo, El Bagre and Zaragoza). The sample size was increased to 50 patients (a 20% increase) to compensate for losses during the follow-up period. This sample size was based on the 1998 WHO protocol [7]. The confidence level was 95% (alpha error = 0.05) and the power of the statistical test was 80% (beta error = 0.20). In 1998, the number of patients with falciparum malaria was 2,200 (total for the three municipalities) [23]. The expected proportion of occurrence of the treatment failure event varies for each treatment and this was, therefore, estimated to 50% for all treatments, in order to obtain a larger sample. The sampling error was fixed to 15%.

### Reference population

The reference population was composed of patients with a history of fever for two to three days, with symptoms compatible with malaria, and a unique infection with *P. falciparum *confirmed by thick smear. These patients attended the malaria diagnosis clinic at their local hospital. Males and non-pregnant females (confirmed by a fast pregnancy test), above one year of age, living in the rural or urban zone of their municipality, where they normally reside, were included in the study.

### Inclusion criteria

Inclusion criteria, besides the features described in "Reference Population" were: a) to have non-complicated malaria (according to a medical evaluation and according to asexual parasitaemia); b) to harbor between 250 and 50,000 asexual parasites/μL; c) exclusion of concomitant disease, after a clinical interview and examination carried out by the research team; d) voluntary participation in the study; e) commitment to attend the follow-up examinations.

### Exclusion criteria

Criteria for exclusion of the study were: a) to evidence, at any moment, severe malaria or any other disease; b) appearance of serious adverse effects due to antimalarial drugs, to other medicines, or to other causes; c) failure to attend any of the first three follow-up examinations (days 1, 2, 3). The evidence of treatment failure to antimalarial therapy resulted in withdrawal from the study and recommendation of a rescue treatment, formulated according to the Colombian Health Ministry guidelines for malaria treatment [24].

### Malaria diagnosis, clinical and parasitological evaluation

Malaria diagnosis was carried out with thick smear according to WHO procedure [25] and routinely used in Colombia. The thick smear and the thin smear were Field- and Giemsa-stained, respectively. The thick smear was examined by microscopy (100×) and the search for parasites was carried out in 200 consecutive microscopic fields. Parasitaemia was calculated on the basis of 200 leucocytes and a constant of 8,000 leucocytes/μL, and was expressed in parasites/μL. A thick smear was diagnosed as negative when no asexual forms were observed in 200 fields. Parasitaemia was reported as asexual parasites/mm^3^, according to 1998 WHO protocol [7].

Each patient, upon admission to the study and at each follow-up, was given: a) a medical evaluation to diagnose the presence of malaria; b) a parasitological evaluation (thick smear, thin smear and parasite count). Special emphasis put on finding, identifying, classifying and defining the origin of adverse effects, which appeared during the 21 to 28 days of follow-up. This search for adverse effects was based on clinical assessment (symptoms, signs), rather than laboratory evaluation. The field team was composed, in each municipality, of a general physician and a clinical laboratory technician, interacting with the local malaria team, a microscopist and a health assistant. An active search for defaulting patients was carried out when needed in order to re-integrate them to the study.

### Treatment

The drugs were taken with water, administered in the doses established by the Colombian Ministry of Health [24] (Table 2). Each treatment was given under supervision by the researchers and the patient was observed during the first half hour. In case of vomiting, the complete dose was supplied again, and the 30-minute observation was repeated. If the patient vomited again, he was excluded and referred to the local hospital. On the last day of follow-up, all patients were administered primaquine, as gametocytocide, in a single dose (0.75 mg/kg; lot C091200).

**Table 2:** Antimalarial drugs administered in eight studies of treatment response. Antioquia (Colombia), 2000–2004 (1)

**Treatment**	**Total Dose**	**Rescue Treatment**
Chloroquine	25 mg/kg: 10 mg/kg day 1; 7.5 mg/kg days 1 and 2	Quinine sulphate – CL
Amodiaquine	25 mg/kg: 10 mg/kg day 1; 7.5 mg/kg days 1 and 2	Mefloquine – SP
Mefloquine	15 mg/kg only one dose on the day 1	Quinine sulphate – SP
Sulphadoxine-pyrimethamine (SP)	25 mg/kg S + 1.25 mg/kg P only one dose day 1	Quinine sulphate – CL
Chloroquine-SP	25 mg/kg: 10 mg/kg day 1; 7.5 mg/kg days 1 and 2	Quinine sulphate – CL
	25 mg/kg S + 1.25 mg/kg P only one dose day 1	
Amodiaquine-SP	25 mg/kg: 10 mg/kg day 1; 7.5 mg/kg days 1 and 2	Mefloquine
	25 mg/kg S + 1.25 mg/kg P only one dose day 1	
Mefloquine-SP	15 mg/kg only one dose day 1	Amodiaquine – SP
	25 mg/kg S + 1.25 mg/kg P only one dose day 1	
Artesunate-SP	12 mg/kg: 4 mg/kg días 0, 1 y 2	Amodiaquine – SP
	25 mg/kg S + 1.25 mg/kg P only one dose day 1	

(1) S sulphadoxine, P pyrimethamine, SP sulphadoxine-pyrimethamine, CL clindamicine.

AQ year 2002 lot 00L22/0439E tab 150 mg Alkem Laboratory; year 2003 lot 3001-ET, Alkem Laboratory.

MQ year 2002 lot 51009 tab 250 mg, Mepha Laboratory; year 2003: lot 350866, Mepha Laboratory.

SP years 2000–2001 lot Sul 50600606; year 2002 lot 09017 RE tab500/25 mg, VAP Laboratory; year 2003–2004: lot RJ0002, Roche Laboratory.

AS lot 351237 Mepha Laboratory.

CQ lot C141100.

QN lot 98C12.

CL lot 000101

### Classification of the treatment response

The follow-up period lasted 28 days in the three groups (AQ, MQ-SP, AS-SP) and 21 days in the five other groups (CQ, MQ, SP, CQ-SP, AQ-SP). TET was classified based on the 1998 WHO protocol [7] with minor changes:

1. Early treatment failure, defined as the presence of any of the following:

a) Development of danger signs or severe malaria on Day 1 Day 2 or Day 3

b) Parasitaemia on day 2 higher than day 0;

c) Parasitaemia on day 3 ≥ 25% of count on day 0.

2. Late treatment failure, defined as the presence of any of the following:

a) Danger signs or severe malaria in the presence of parasitaemia (same species of day 0) after day 3;

b) Non-programmemed return of the patient between days 4 to 21 (CQ, SP, CQ+SP) or 28 (for other), due to a clinical deterioration in the presence of parasitaemia (same species of day 0).

c) Parasites (same species of day 0) on days 7, 14, 21, or 28.

3. Adequate clinical-parasitological response: if the patient did not present either early or late failures.

### Data processing and statistical analysis

The EpiInfo 6.04 programmeme was used to make questionnaires and data bases and also to analyse information (Centers for Disease Control and Prevention, USA; World Health Organization, Geneva, Switzerland. EpiInfo 6.04). SPSS 10.0 was also used for data analysis and graph construction.

Associations among groups or independent variables (such as treatment schedules, type of treatment response) were measured with square chi test (χ^2^); the means were compared with Wilcoxon or Kruskal-Wallis (K-W) tests. A significance level of 5% (α = 0.05) was always established.

The following abbreviations and expressions were used: n = number of patients, X = mean or average or arithmetic mean, Me = median, SD = Standard deviation, SE = Standard error, P25 = percentile 25, P75 = percentile 75, IC95% = confidence interval of 95% for a determined value, such as the mean; LL and HL are lower or high limits of IC95%, p = probability.

### Ethical approval

Written informed consent from each patient or guardian of an underage child, was obtained. Each project was approved by the Ethics Committee and by the Center of Medical Investigations (CIM) of Universidad de Antioquia.

## Results

Of the 520 patients included in the study, 47% were recruited in Turbo (Urabá zone) and 53% in The Bajo Cauca zone (El Bagre 37% and Zaragoza 16%). Data were analysed independently from the residence place of the patient since important differences were not found when this variable was considered.

The patients exhibited the following characteristics:

a) 61% were living in the rural zone

b) 69% were men

c) 29% were under 15 years of age (3% <5, 11% 5–9 years, 15% 10–14 years, 1% were older than 64 years, and the remaining 70% corresponded to patients from 15 to 64 years old (34% 15–24, 27% 25–44 and 9% 45–64 years).

TET could be measured in 93.7% of the patients (487 out of 520 patients). The reason for not evaluating TET in 33 patients was the impossibility of finding them (90%: 30/33) or voluntary withdrawal from the investigation (10%: 3/33). Failure to attend increased further into the follow-up time. Such defaulting was statistically different among treatments, and the four monotherapies had less defaulting overall (4%) than the four treatment combinations (8%). This percentage of defaulting per treatment varied from major to minor: a) in monotherapies: it was 9% with MQ, 3% with AQ, none with CQ and SP; b) in combinations: it was 12% with MQ-SP, 10% with AQ-SP and AS-SP, none with CQ-SP. Defaulting was higher (9.3%) in El Bagre-Zaragoza, which largely depends for communication on the Nechí River, than in Turbo (5.7%), in which basic transport is by land. The group of 33 non-attending patients did not differ from the non-defaulters (n = 487) in relation to the residence zone (urban, rural), sex, ethnicity, age, days of evolution of the current disease and initial parasitaemia.

The mean of asexual parasitaemia in 487 patients before treatment administration was 7,700 parasites/μL, the median was 4,480, the 25 percentile was 2,000; the 75 percentile was 10,130 and the standard deviation was 8,472 parasites/μL. A 26% of patients had as many as 2,000 parasites/μL, 45% as many as 4,000, 50% up to 5,000, 75% up to 10,000 and only 8% over 20,000 parasites/μL.

The mean asexual parasitaemia before treatment administration was not statistically equal among the treatments; it varied between 8,954 (MQ) and 5,203 parasites/μL (CQ-SP) (p < 0.05); between MQ and SP (5,663 parasites/μL) there was a statistically significant difference. Gametocytes were found in 17% of patients before treatment, in 23% on day 1, 33% on day 2, 43% on day 3, 65% on day 7, 64% on day 14, 51% on day 21, and 15% on day 28. Before the treatment, the mean number of gametocytes ranged from 22 (AS-SP) to 140 gametocytes/μL (AQ-SP), with a general mean of 78 and a standard deviation of 421 gametocytes/μL (minimum 0 and maximum 7,600).

Monotherapy with CQ showed 82% failure, with AQ it showed 30%, with MQ 4%, and with SP 24%, while the combinations of SP with other drugs showed the following failure rates: CQ-SP 17%, AQ-SP 2%, MQ-SP 0% and AS-SP 3% (Table 4). There were 62 failures (12,7%) in 487 patients in whom the final effect could be measured. Early failure contributed with 42% of the total failure (26/62) and the late failure accounted for 58% (36/62) of it. Considering defaulting patients as failures the percentage of failure reached 18% (62 observed failures + 30 supposed failures/520). The frequency of early and late failures changes from one treatment to another: a) with monotherapies, early and late failures were equal to the those with CQ, early failures were predominant with AQ (ratio 1:4), and the late ones were the majority with SP (2:1); b) with the CQ-SP combination, the late failures widely prevailed (9:1). With the rescue treatment, which varied according to the primary treatment, all treatment responses were successful; which meant no failure of two treatments was observed.

Treatment failure was not associated with initial asexual parasitaemia (pre-treatment), although there were important differences as follows: those who successfully responded and those who had early failure had 7,760 and 7,951 parasites/μL, respectively, while those who had late failure showed 6,731 parasites/μL; despite that, the differences were not statistically significant. Neither was there any relation of TET with initial gametocytaemia. Thus, although initial asexual parasitaemia was not equal in all eight treatments, this fact did not have any effect in the TET.

It should be pointed out that there was no significant association of the variable TET with any one of these explicative variables: municipality, zone, sex, age group, ethnic group, malaria antecedents, and hospitalization or transfusion antecedents.

## Discussion

Antimalarial schemes administered (dose, scheme, application time) corresponded to those used for many years in Colombia and recommended by other authors [8,26]. Direct surveillance of patients by the researchers during drug intake assured that the drugs were taken, and ruled out losses due to vomiting or diarrhea.

The protocol used for measuring TET was identical in most studies, with exception of the studies of 2000–2001, when CQ, SP and CQ-SP were evaluated over 21 days; in those undertaken between 2001 and 2004, the follow-up lasted 28 days.

The number of patients in whom TET could not be measured (lost patients) was low (6% in the eight studies) and was not affected by the minimum sampling size. The strategy used to avoid defaulting consisted of monetary support (of up to 5,000 Colombian pesos, i.e. less than US$2) to cover transportation costs, as well as the Immediate Active Search (IAS) for any patient who missed a follow-up examination. In fact, defaulting rate has been higher than 40%, but was reduced to 6% through the application of that strategy. IAS was performed by the physician or clinical laboratory technician, accompanied by the health assistant, using motorcycles supplied by the local hospital. Even in the worst case scenario, i.e. if all patients in whom TET was could not be measured are considered as failures, the percentage of treatment failure for each treatment schedule was under 14%, except in the case of AQ, which had net failure (excluding defaulters) of 30%, and gross failure of 33% (when counting defaulters as failures).

From the evaluated schizonticide monotherapies (CQ, AQ, MQ, SP), only CQ has been used in this way in Colombia, although associated to primaquine as gametocide (45 mg in a single dose). CQ had become useless in 1998 (according to López *et al*. [27] and Blair *et al *[28-30]), due to recorded failures between 71% and 67% now reaching 82%. The national average of CQ failure from 1961–2003 is 66% (Table 1), which indicates that it should no longer be used anywhere in the country. The CQ-SP combination (failure 17%) seems to remains useful, but efficacy should be evaluated in other areas (e.g. Orinoquia, Amazonia, Catatumbo).

The accelerated growth of failure presented by the monotherapy AQ is serious because it increased from 7% in 1998 in El Bagre [27], Zaragoza [28,30] and Turbo [28,29] to 30% in 2004, which excludes the drug for monotherapy, according to the 25% threshold proposed by WHO [7]. The national average of AQ failure is 22%, but there are recent data (2002–2003) coming from Tadó (Chocó) indicating a failure rate of 27% (Table 1) and from Tumaco (Nariño) which reports 50%, although there was no failure in El Charco, in the same state [31].

Failure of the monotherapy with SP also increased strongly, going from 12% in 1998 [27-30] to 24% current. The MQ-SP combination showed a zero failure rate, but AS-SP showed a failure of 3%, although never officially used in Colombia.

The AQ-SP combination is highly efficient (98%). The relatively scantly use of the AQ was a result of the adverse reactions which had been observed when AQ was used for prophylaxis [32], a situation in which it is taken for weeks or months, but reports on serious adverse effects of its use as an antimalarial are practically absent [33-35]. In a different study carried out by us in Turbo, 57 patients treated with AQ monotherapy were clinically and laboratory (blood and liver tests) assessed before treatment and 10 days after it. We concluded that, at the standard dose (25 mg/kg peso) and period (3 days), as recommended for uncomplicated falciparum malaria, AQ had no adverse effects or toxicity (paper in preparation).

A systematic review of 2001 compared AQ-SP and CQ-SP combinations for the treatment of non-complicated malaria; on the basis of seven studies in 1,277 patients, it concluded that there was no evidence of serious collateral effects with those combinations and that they, more than monotherapy with SP, "could make people feel better more rapidly", and caused a better steady elimination of blood parasites [36]. In 2005, a Cochrane review aimed at assessing " the combination of CQ or AQ plus SP compared with SP alone for first-line treatment of uncomplicated falciparum malaria", concluded that "the evidence base is not strong enough to support firm conclusions. The available evidence suggests that AQ plus SP can achieve less treatment failure than SP, but this might depend on existing levels of parasite resistance to the individual drugs" [37].

According to the results obtained in this study and to the additional information presented here, treatment with AQ-SP should remain as the first line treatment of non-complicated falciparum malaria in Colombia, as it is at present [24], leaving options based on MQ and on AS for the future, once AQ-SP has failed.

Colombia should establish an "Antimalarial Failure Epidemiological Surveillance System" (AFESS). The AFESS should have, as primary objective, the early detection of failure by means of a screening test. For this, a selected screen test should be low cost, applicable in field conditions and have a high sensitivity. Our recommendation is to install an AFESS coupled to the antimalarial programmeme, to measure TET during a following up period of 7 – 14 days and to install sentinel posts in strategic places of malaria regions. In addition the AFESS, should: a) carry out specific research projects aimed at confirmation of therapeutic failure after a following up period of 28 days or more, b) evaluation of *P. falciparum in vitro *resistance; c) *P. falciparum *y *P. vivax *genetic analysis and d) assessment of antimalarial blood concentrations in patients studied.

**Table 3:** Treatment response according to antimalarial treatment; Antioquia (Colombia), 2000–2004 (1)

**Response **(2)	**CQ**	**AQ**	**MQ**	**SP**	**CQ-SP**	**AQ-SP**	**MQ-SP**	**AS-SP**	**Total**
Early Failure	8	2	1	12	1	1	0	1	26
%	***47.1***	***6.1***	***1.1***	***15.2***	***1.6***	***1.1***	***0.0***	***1.7***	***5.3***
Late Failure	6	8	3	7	10	1	0	1	36
%	***35.3***	***24.2***	***3.3***	***8.9***	***15.6***	***1.1***	***0.0***	***1.7***	***7.4***
Total Failure	14	10	4	19	11	2	0	2	62
%	**82.4**	**30.3**	**4.4**	**24.1**	**17.2**	**2.2**	**0.0**	**3.4**	**12.7**
Adequate	3	23	88	60	53	88	55	55	425
%	***17.6***	***69.7***	***95.6***	***75.9***	***82.8***	***97.8***	***100.0***	***96.6***	***87.3***
Total	17	33	92	79	64	90	55	57	487
%	100.0	100.0	100.0	100.0	100.0	100.0	100.0	100.0	100.0

(1) The eight schemes were assessed in a sequential way, between 2000 and 2004.

(2) Failures %: Total failures (early failures + late failures)/patients with measured antimalarial treatment response; it means, non counting the lost patients.
